# A mixed methods study of perinatal depressive and anxiety symptom prevalence and the role of traditional healing alongside clinical care in Southern Malawi

**DOI:** 10.1007/s44192-026-00554-w

**Published:** 2026-07-29

**Authors:** Omero G. Mwale, Japhet Myaba

**Affiliations:** 1 Department of Community and Mental Health, The Catholic University of Malawi, Limbe, Malawi; 2 Department of Nursing Sciences, Kamuzu University of Health Sciences, Blantyre, Malawi

**Keywords:** Traditional medicine, Clinical care, Perinatal depressive and anxiety symptoms, Malawi, Mixed methods

## Abstract

**Background:**

Perinatal mental health remains a critical yet insufficiently addressed component of perinatal care in sub-Saharan Africa. In Southern Malawi, where cultural beliefs and traditional healing practices profoundly shape health-seeking behaviour, the intersection of traditional and clinical care presents both challenges and opportunities for improving perinatal outcomes.

**Objective:**

This study investigated the prevalence of perinatal depressive and anxiety symptoms and explored the role of traditional healing practices alongside clinical care in supporting perinatal mental health in Southern Malawi.

**Methods:**

A convergent parallel mixed-methods design was employed. Quantitative data were collected from perinatal mothers (n = 330) using the Patient Health Questionnaire-9 (PHQ-9) and the Generalized Anxiety Disorder-7 (GAD-7) scale. Qualitative data were gathered through semi-structured interviews with perinatal mothers (n = 10), healthcare providers (n = 10), and traditional healers (n = 10). Quantitative data were analysed with SPSS using descriptive statistics; qualitative data were analysed using Braun and Clarke’s reflexive thematic analysis framework.

**Results:**

Approximately 35% of perinatal mothers screened positive for moderate-to-severe depressive symptoms on the PHQ-9 (mean score: 10.5, SD = 4.3), while 30% screened positive for moderate-to-severe anxiety symptoms on the GAD-7 (mean score: 9.2, SD = 3.9). These findings reflect validated self-reported screening results and should not be interpreted as clinical diagnoses. Sixty percent of mothers relied on traditional healing methods for perinatal mental health concerns, citing cultural familiarity and trust. Forty percent faced barriers to clinical care, including stigma and limited awareness of available services. Qualitative data revealed that traditional healers provided essential emotional support and community cohesion, while healthcare workers reported inadequate training in perinatal mental health assessment and intervention.

**Conclusion:**

The integration of traditional and clinical practices represents a potentially feasible strategy for enhancing perinatal mental health outcomes in Southern Malawi. Findings highlight the urgent need for structured training of healthcare providers, community-sensitive interventions, and collaborative frameworks that leverage the complementary strengths of both healing systems. These insights are relevant to other low-resource settings across sub-Saharan Africa.

## Introduction

Perinatal mental health is a pressing yet frequently overlooked component of comprehensive perinatal care worldwide. Conditions such as perinatal depression and anxiety affect a substantial proportion of women during pregnancy and the postpartum period, with global prevalence estimates ranging from 10 to 25% [1, 2]. Sub-Saharan Africa bears a disproportionate share of this burden, where the pooled prevalence of postpartum depressive symptoms ranges from 3.8 to 69.9%, with an estimated 22.1% of women affected [3]. Poor perinatal mental health undermines caregiving capacity, adversely affects infant development, and disrupts family dynamics, creating a cycle of disadvantage that extends well beyond the individual mother [1].

In Malawi, previous research has reported perinatal depression rates of 10.7–25.8% during pregnancy and in the postpartum period [4]. These challenges are compounded by poverty, limited healthcare access, inadequate mental health infrastructure, and pervasive social stigma [5].

Traditional healing practices are deeply embedded in Malawian culture and serve as the primary source of healthcare for approximately 80% of the population, particularly in rural areas [6, 7]. Traditional healers provide culturally congruent care that addresses physical, emotional, and spiritual dimensions of well-being through practices rooted in local beliefs and customs [8]. In the context of perinatal mental health, these practitioners often function as first-line providers, offering support that is both accessible and culturally meaningful [9].

Perinatal healthcare in Southern Malawi is delivered primarily through a tiered public health system comprising community health centres, rural and community hospitals, and a small number of district and central hospitals, with antenatal and postnatal care largely nurse- and midwife-led. Formal mental health services remain concentrated at central hospital level, with very few facilities at primary or secondary level offering routine perinatal mental health screening or psychological support. Malawi’s national mental health policy framework and the Mental Health Act (2022) recognise mental health as a priority area and call for integration of mental health into primary care, but implementation at community level, particularly for perinatal populations, remains limited, with persistent shortages of trained mental health personnel and screening protocols within maternal and child health services [8, 9].

Traditional healing in Malawi encompasses several distinct, though sometimes overlapping, categories of practitioners: herbalists (“asing’anga”), who use plant- and root-based remedies; spiritual or faith healers, who address illness through ritual and spiritual intervention; traditional birth attendants, who support pregnancy, labour, and the immediate postpartum period; and diviners, who are consulted to identify the underlying cause of illness, including perceived spiritual or interpersonal causes. For consistency, the term “traditional healer” is used throughout this manuscript to refer collectively to these practitioner types, with specific sub-categories noted where relevant to participants’ accounts.

Despite this widespread utilisation, the integration of traditional healing with clinical mental health services remains limited. Barriers to clinical care include the absence of mental health services within primary healthcare facilities, a critical shortage of mental health professionals, insufficient resource allocation, and significant societal stigma [9]. Differing etiological frameworks — whereby clinical models emphasise biomedical explanations while traditional perspectives frequently attribute mental illness to spiritual causes or interpersonal conflict,further complicate efforts at integration [8].

Against this background, this study aimed to explore how traditional and clinical approaches coexist in Southern Malawi, and how they may be synergised to achieve better perinatal mental health outcomes. Specifically, the study was guided by four objectives: (1) to assess the prevalence of perinatal depressive and anxiety symptoms; (2) to explore the role of traditional healing practices in perinatal mental health care; (3) to describe barriers to accessing clinical mental health services among perinatal mothers; and (4) to investigate potential strategies for integrating traditional healing with clinical care.

Findings are intended to inform policy and practice in Southern Malawi and comparable low-resource settings across Africa.

### Conceptual framework

**Fig. 1:**
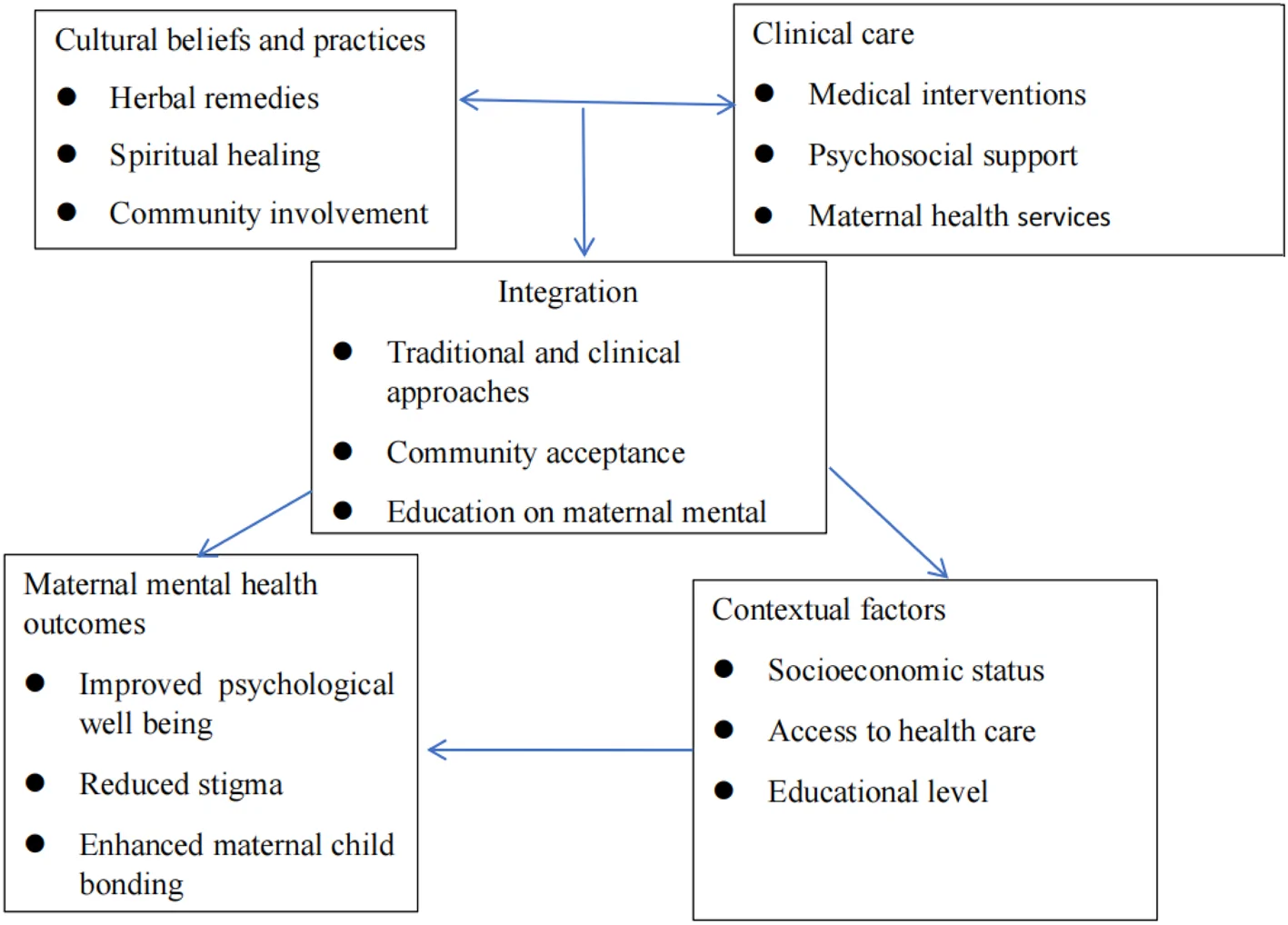
Belief model—conceptual framework underpinning this study

The study was guided by the Belief Model (Fig. 1), which provides a conceptual basis for understanding how cultural beliefs and biomedical care interact to influence perinatal mental health outcomes.

The model posits that traditional healing practices (including herbal remedies, spiritual interventions, and community-based support) and clinical care (comprising medical and psychological interventions) operate in parallel but can be effectively integrated. This integration promotes community acceptance of clinical services by respecting and acknowledging cultural practices, while education on perinatal mental health bridges existing gaps in care-seeking behaviour. The resulting interaction is proposed to improve psychological well-being, reduce stigma, and strengthen the mother–infant bond. Contextual factors, including socioeconomic status, educational attainment, and geographic access to services, moderate the effectiveness of each approach and must be considered when designing integrated interventions.

### Overview of related literature

Several studies have documented the burden of perinatal depressive and anxiety symptoms in Malawi and the broader sub-Saharan African region. Chilanga and colleagues [10] demonstrated significant rates of postpartum depression in both urban and rural settings in Malawi, emphasising the need for targeted interventions. Mphande and Chisale [11] similarly identified high prevalence of anxiety and depression among perinatal mothers, with low socioeconomic status and lack of social support as major risk factors. The comparative analysis by Kakoma and colleagues [12] further highlighted regional variability, suggesting that geographical and cultural factors shape perinatal mental health outcomes for perinatal women.

Regarding traditional healing, Phiri and colleagues [13] found that many women in Malawi rely on traditional healers as primary mental health support providers, with these practices often complementing rather than replacing clinical care. Qualitative work by Mwale and Banda [14] demonstrated that traditional birth attendants frequently serve as the first point of contact for perinatal mental health concerns. Mkhabela and colleagues [15] noted that women generally regard traditional healers as trustworthy and culturally appropriate, though better integration with clinical services remains necessary.

Barriers to clinical mental health care are well documented. Kachale and colleagues [16] identified stigma, lack of awareness, and logistical challenges as primary obstacles, while Chibwana and Mwansambo [17] drew attention to systemic gaps in infrastructure and mental health literacy. Stigma in particular has been shown to significantly deter mothers from accessing formal services [18, 19]. Integration frameworks proposed by Chilima and Chirwa [20], Phiri and colleagues [21], and policy recommendations advanced by Manda and Ngwira [22] underscore the potential of community-based, culturally sensitive models that engage both traditional healers and clinical practitioners.

Despite this growing evidence, there remains a paucity of mixed-methods research that jointly quantifies perinatal depressive and anxiety symptom prevalence and qualitatively explores the role of traditional healing in the same cohort within a low-resource African setting. This study addresses that gap by adopting a convergent parallel design in Southern Malawi, where both traditional and clinical care systems are actively utilised.

## Methods

This study received ethical approval from the Catholic University of Malawi Research Ethics Committee (see Declarations section for full details). No external funding was received for this study. The methods below were conducted in accordance with the approved protocol.

### Study design and setting

This study employed a convergent parallel mixed-methods design [23], in which quantitative and qualitative data were collected concurrently, analysed independently, and subsequently merged to produce an integrated interpretation. The research was conducted in selected health centres, community villages, and a district hospital in Southern Malawi.

### Participants

Three participant groups were recruited: (1) perinatal mothers who had accessed both traditional and clinical care, (2) traditional healers formally recognised within their communities, and (3) healthcare providers employed at participating facilities. This diverse sample was designed to capture multiple perspectives on perinatal mental healthcare.

### Sampling and sample size

#### Perinatal mothers

Given that prevalence estimation was a primary study outcome, the sampling strategy for perinatal mothers is described in full here. The sampling frame comprised perinatal women (antenatal and postnatal) attending the participating health centres, community postnatal clinics, and the district hospital in the selected Southern Malawi district during the study period. A cluster-based approach was used: health facilities and their catchment community clinics served as primary sampling clusters, from which eligible women were recruited consecutively during routine visits until the target sample was reached. Eligibility screening required that women were (i) aged 18 years or older, (ii) currently pregnant or within 12 months postpartum, (iii) resident in the catchment area, and (iv) had accessed both traditional and clinical care for any perinatal health concern, as confirmed through a brief eligibility interview prior to enrolment. Because recruitment was facility- and cluster-based rather than drawn from a full population sampling frame, and because eligibility was restricted to dual-care users, the resulting prevalence estimates should be interpreted as reflecting this defined sub-population of perinatal women in the study catchment area rather than the general population of perinatal women in Southern Malawi (see Limitations, Sect. 4.1).

The required sample size for the quantitative component was calculated using the standard formula for cross-sectional prevalence studies:

$$ n = Z^{2} \times p \times \left( {1 - p} \right){/}E^{2} $$

where Z = 1.96 (95% confidence level), *p* = 0.50 (assumed maximum variability), and E = 0.05 (margin of error), yielding n = 384. To maintain feasibility while ensuring adequate representation and accounting for potential non-response, the target sample was set at 350. Ultimately, 330 completed questionnaires were returned, representing a response rate of 94.3%.

Participants were recruited by trained research assistants at participating health centres and community postnatal clinics during routine perinatal visits between June and August 2025. Research assistants approached eligible women directly, explained the study’s purpose and voluntary nature, and obtained written informed consent before enrolment. No financial incentives were offered; light refreshments were provided during interviews at community venues as standard practice in community-based research in this context.

#### Traditional healers and healthcare providers

Purposive sampling was used to select ten traditional healers based on the following operationalised criteria: (1) formal recognition as a traditional healer by the local chief or community health committee, (2) a minimum of five years of active practice, and (3) documented experience providing care to women of reproductive age. Ten healthcare providers were recruited through convenience sampling from participating facilities. For both groups, data collection continued until thematic saturation was achieved,meaning no new substantive themes emerged from additional interviews [24]. Following an initial round of ten interviews per group, two supplementary interviews were conducted per group to confirm saturation.

### Data collection

#### Quantitative instruments

Mental health status among perinatal mothers was assessed using two validated instruments. The Patient Health Questionnaire-9 (PHQ-9) measures the severity of depressive symptoms across nine items rated on a four-point scale (0 = ‘not at all’ to 3 = ‘nearly every day’), with scores categorised as minimal/no depression (0–4), mild (5–9), moderate (10–14), moderately severe (15–19), and severe (20–27) [25]. The Generalized Anxiety Disorder-7 (GAD-7) assesses anxiety severity across seven items using the same response scale, with scores categorised as minimal/no anxiety (0–4), mild (5–9), moderate (10–14), and severe (15–21).

Both instruments were translated into Chichewa (the local vernacular) to enhance comprehension. Trained research assistants administered them alongside structured closed-ended questionnaires addressing healthcare utilisation, satisfaction, and help-seeking behaviour. Data collection occurred at community health centres and during postnatal visits.

#### Qualitative interviews

Semi-structured interviews were conducted with all three participant groups: perinatal mothers (n = 10), traditional healers (n = 10), and healthcare providers (n = 10). Interviews were guided by flexible, open-ended prompts covering broad areas of interest, including: the role of traditional and clinical practices in perinatal mental healthcare; perceived barriers to service access; cultural explanatory models of perinatal mental illness; and potential strategies for integration. No themes were predetermined; all themes were generated inductively from the data following reflexive thematic analysis. Interviews were conducted in the local language by trained interviewers, audio-recorded with participant consent, and subsequently transcribed and translated.

### Data analysis

#### Quantitative analysis

Quantitative data were entered into IBM SPSS Statistics (Version 25) following cleaning and checking for missing values and outliers. Descriptive statistics were calculated to characterise the sample and report prevalence rates based on established PHQ-9 and GAD-7 clinical thresholds (Tables 1, 2, 3 and 4).

**Table 1:** Demographic characteristics of perinatal mothers (n = 330)

Characteristic	Category	n	%
Age group	18–24 years	92	27.9
	25–34 years	224	67.9
	≥ 35 years	14	4.2
Socioeconomic status	Low income	231	70.0
	Middle income	83	25.2
	High income	16	4.8
Education level	No formal education	66	20.0
	Primary education	145	43.9
	Secondary education	99	30.0
	Tertiary/higher education	20	6.1
Perinatal period	Antenatal	181	54.8
	Postnatal	149	45.2

**Table 2:** PHQ-9 and GAD-7 score distributions among perinatal mothers

Instrument/severity	Score range	n	%	Mean (SD)
PHQ-9 (Depression)				10.5 (4.3)
Minimal/none	0–4	115	34.8	
Mild	5–9	99	30.0	
Moderate	10–14	66	20.0	
Moderately severe	15–19	33	10.0	
Severe	20–27	17	5.2	
GAD-7 (Anxiety)				9.2 (3.9)
Minimal/none	0–4	132	40.0	
Mild	5–9	99	30.0	
Moderate	10–14	59	17.9	
Severe	15–21	40	12.1

**Table 3:** Profile of healthcare worker participants (n = 10)

ID	Age	Qualification	Exp. (yrs)	Role
HW01	32	Certificate in Nursing	5	Staff Nurse, District Hospital
HW02	45	Bachelor of Science in Nursing	15	Nursing Officer, Health Centre
HW03	38	Certificate in Nursing	8	Staff Nurse, District Hospital
HW04	29	Certificate in Midwifery	2	Midwife, Health Centre
HW05	52	Bachelor of Science in Nursing	20	Senior Nursing Officer
HW06	41	Certificate in Nursing	12	Staff Nurse, District Hospital
HW07	35	Certificate in Midwifery	7	Midwife, Community Centre
HW08	48	Bachelor of Science in Nursing	18	Nursing Officer, Health Centre
HW09	27	Certificate in Nursing	2	Staff Nurse, Health Centre
HW10	43	Bachelor of Science in Nursing	13	Senior Nursing Officer

**Table 4:** Profile of traditional healer participants (n = 10)

ID	Age	Training	Exp. (yrs)	Specialisation
TH01	55	Informal/herbal medicine certificate	25	Herbal remedies and spiritual healing
TH02	42	Informal apprenticeship	10	Maternal health and midwifery
TH03	60	Informal/herbal medicine certificate	30	Mental and spiritual healing
TH04	48	Informal apprenticeship	15	Community healing practices
TH05	35	Herbal medicine certificate	5	Women’s health and childbirth
TH06	50	Informal/herbal medicine certificate	20	Anxiety and emotional support
TH07	38	Informal apprenticeship	8	Maternal and child health
TH08	57	Informal/herbal medicine certificate	28	Spiritual and community healing
TH09	30	Herbal medicine certificate	5	Maternal health
TH10	44	Informal apprenticeship	12	Community and mental health

#### Qualitative analysis

Qualitative data were analysed using Braun and Clarke’s [26] six-phase reflexive thematic analysis framework: (1) familiarisation with the data; (2) systematic coding; (3) generation of initial themes; (4) review and refinement of themes; (5) defining and naming themes; and (6) producing the final report with representative quotations. Codes were generated inductively from the data and applied consistently across all three participant groups to ensure a comprehensive, cross-cutting interpretation (Table 5).

**Table 5:** Themes and sub-themes from qualitative analysis with illustrative quotations

Theme	Sub-themes	Illustrative quote
1. Cultural trust in traditional healing	Cultural familiarity; perceived effectiveness; community belonging	“I feel more understood by my traditional healer. They speak my language and know my struggles.”—Perinatal mother
2. Barriers to clinical mental health services	Stigma; lack of awareness; inadequate infrastructure; dismissive attitudes	“When I went to the hospital, I felt like they didn’t take my feelings seriously. I wanted to talk, but they were more focused on my physical health.”—Perinatal mother
3. Role of traditional healers in emotional support	Emotional reassurance; community integration; addressing broader social needs	“When a woman comes to me, I do not only treat her body—I try to understand her heart and her family situation.”—Traditional healer
4. Knowledge gaps among healthcare providers	Inadequate training; limited screening; under-recognition of perinatal depressive and anxiety symptoms	“During our training we never learnt how to assess mothers with perinatal mental health problems. We need a short training on this.”—Healthcare worker
5. Potential for integration of care	Complementary roles; mutual referral; community acceptance of clinical care	“If the clinic and the healer could work together, many mothers would benefit—we each see different things.”—Healthcare worker

### Trustworthiness

Several strategies were employed to enhance the rigour of the qualitative findings. Credibility was supported through methodological triangulation (concurrent quantitative and qualitative data collection) and member checking, whereby emerging findings were reviewed by a subset of participants [27]. Transferability was addressed by providing rich contextual descriptions of the study setting and participant profiles [28]. Dependability was ensured through a detailed audit trail and regular consultations with co-researchers [24]. Confirmability was promoted through researcher reflexivity [29] and peer-debriefing sessions [30].

### Ethical considerations

Ethical approval was obtained from the Catholic University of Malawi Research Ethics Committee in accordance with the revised Declaration of Helsinki. Written informed consent was obtained from all participants prior to data collection. Confidentiality and anonymity were maintained throughout. Participants were informed of their right to withdraw at any time without consequence.

## Results

### Demographic characteristics

A total of 330 perinatal mothers, 10 healthcare workers, and 10 traditional healers participated in the study. Most perinatal mothers were aged 25–34 years (67.9%), with 27.9% aged 18–24 and 4.2% aged 35 years or older. Seventy percent were from low-income socioeconomic backgrounds, and 43.9% had attained primary education only, reflecting the limited educational opportunities prevalent in the study setting. Full demographic details are presented in Table 1.

Healthcare worker profiles are described in Table 3; this group demonstrated diverse educational qualifications ranging from nursing certificates to bachelor’s degrees and clinical experience of 2–20 years. Traditional healers (Table 4) predominantly held informal training or herbal medicine certificates, with experience ranging from 5 to 30 years and ages between 30 and 60 years.

### Prevalence of depression

Analysis of PHQ-9 scores revealed that 35.2% of participants reported moderate-to-severe depressive symptoms. Specifically, 20.0% met the criteria for moderate depression (PHQ-9 = 10–14), 10.0% for moderately severe depression (PHQ-9 = 15–19), and 5.2% for severe depression (PHQ-9 ≥ 20). The mean PHQ-9 score was 10.5 (SD = 4.3). Full score distributions are shown in Table 2. It is important to note that these figures reflect self-reported symptom screening using PHQ-9 cut-off scores and do not constitute clinician-confirmed diagnoses of depressive disorder.

### Prevalence of anxiety

GAD-7 results indicated that 30.0% of participants exhibited moderate-to-severe anxiety. Of these, 17.9% scored in the moderate range (GAD-7 = 10–14) and 12.1% in the severe range (GAD-7 ≥ 15). The mean GAD-7 score was 9.2 (SD = 3.9). As with the PHQ-9 findings, GAD-7 scores indicate screening-level symptom severity rather than a formal clinical diagnosis of an anxiety disorder. These prevalence rates nonetheless represent a substantial public health burden warranting further attention and follow-up clinical assessment.

### Healthcare-seeking behaviour

Approximately 60% of perinatal mothers reported utilising traditional healing methods for perinatal mental health concerns. Women who chose traditional healing cited cultural familiarity and trust in the healer as the primary determinants. The remaining 40% sought care from clinical healthcare providers; however, this group reported encountering significant barriers, including stigma, limited awareness of available mental health services, and unhelpful encounters with clinical staff who focused predominantly on physical health.

### Qualitative findings

#### Role of traditional healing

Qualitative interviews with both traditional healers and perinatal mothers revealed that traditional healers occupy a central and multifaceted role in the lives of perinatal women. According to traditional healers and healthcare providers interviewed, beyond addressing mental health symptoms, traditional healers provided emotional reassurance, fostered a sense of community belonging, and often mediated family and relational difficulties perceived as contributing to perinatal distress. A perinatal mother stated: “*I feel more understood by my traditional healer. They speak my language and know my struggles*.” A traditional healer similarly observed: “When a woman comes to me, I do not only treat her body, *I try to understand her heart and her family situation.”*

#### Barriers to clinical mental health care

Participants consistently identified a cluster of barriers that prevented engagement with clinical mental health services. Stigma emerged as the most prominent, with many women fearing social judgment if they disclosed mental health difficulties. Lack of awareness about the availability and legitimacy of perinatal mental health care was also widely reported. Structural barriers included limited service availability in rural areas, distance to facilities, and long waiting times. A perinatal mother summarised a common experience: “*When I went to the hospital, I felt like they didn’t take my feelings seriously. I wanted to talk, but they were more focused on my physical health*.”

#### Knowledge gaps among healthcare providers

Healthcare workers acknowledged significant deficiencies in their training related to perinatal mental health care and services. None reported having received formal instruction in the use of standardised screening tools or in the management of perinatal depression and anxiety. One healthcare worker stated: “During our training, we never learnt how to assess mothers with perinatal mental health problems. We need a short training on this.” This finding points to a critical systemic gap that undermines the quality of perinatal care.

## Discussion

This study adds to a growing body of evidence on the substantial burden of perinatal depressive and anxiety symptoms in Malawi and the complex interplay between traditional healing and clinical care in this context. The finding that 35% of perinatal mothers exhibited moderate-to-severe depressive symptoms and 30% demonstrated moderate-to-severe anxiety is consistent with previously reported rates in sub-Saharan Africa and underscores the urgency of the problem [1, 3, 4]. These figures are particularly concerning given that the majority of affected women are from low-income households with limited formal education factors known to exacerbate vulnerability to perinatal mental illness and to impede access to appropriate care [5].

The predominance of traditional healing as the preferred source of perinatal mental health support (60% of participants) is consistent with wider literature documenting the centrality of traditional medicine in Malawian healthcare-seeking behaviour [6, 7]. This figure aligns with national estimates suggesting that the majority of Malawians rely on traditional medicine as a primary source of healthcare, indicating that our sample reflects broader population patterns. Importantly, women’s preference for traditional healers was not rooted in a rejection of biomedicine but in the cultural congruence and relational trust that traditional healing provides, attributes that are often absent in under-resourced clinical settings [31]. This finding mirrors that of Phiri and colleagues [13] and van der Geest [32], who highlight the indispensable role of culturally rooted practices in perinatal mental health care for women in low-income African settings.

It is important to note that the study recruited only women who had accessed both traditional and clinical care. Women who relied exclusively on one system may exhibit different perinatal mental health profiles, and the prevalence figures reported here should be interpreted within this context. Future studies should include women who access only traditional care or only clinical care in order to provide a more complete epidemiological picture.

The barriers identified by participants,particularly stigma, lack of awareness, and inadequate infrastructure—align closely with findings from Kachale and colleagues [16], Chibwana and Mwansambo [17], and Phiri and colleagues [18]. These barriers collectively constitute a system-level failure to prioritise mental health within perinatal care, contributing to a pattern in which clinically significant psychological distress goes undetected and untreated. The dismissive attitudes reported by participants in clinical encounters represent an additional layer of structural harm that reinforces stigma and deters future help-seeking [33, 34].

A particularly important finding is the inadequacy of healthcare worker training in perinatal mental health care and services. Providers openly acknowledged that their pre-service education had not equipped them with the knowledge or skills to screen for or manage perinatal depression and anxiety. This gap has important downstream consequences: without trained frontline staff capable of identifying perinatal distress, the opportunity for early intervention is lost, and the burden falls disproportionately on traditional healers who lack clinical support resources [35, 36]. Evidence from comparable settings confirms that targeted in-service training in mental health can significantly improve provider knowledge, screening practices, and referral behaviour [2].

Regarding traditional healers, the study highlights the absence of formal training in perinatal mental health among these practitioners. While their cultural knowledge and community trust are valuable assets, the data suggest that traditional healers currently operate without structured frameworks for recognising clinical-level symptoms or for facilitating appropriate referrals. Structured capacity-building programmes that build on their existing strengths could significantly enhance their ability to identify women at risk and to refer them for clinical assessment, thereby strengthening the continuum of care [9].

The qualitative data provide nuanced insights into the complementary potential of the two systems. While traditional healers offer emotional attunement, community embeddedness, and holistic contextualisation of distress, clinical care offers diagnostic precision, pharmacological management, and evidence-based psychological therapies. Neither system alone is adequate; together, they have the potential to provide a more comprehensive response to perinatal mental health needs. This is consistent with the integration frameworks proposed by Chilima and Chirwa [20], the policy recommendations of Manda and Ngwira [22], and the community-based integration models described by Zimba and Chirwa [37], all of which advocate for structured collaboration.

Taken together, these findings point to three priority areas for action. First, culturally sensitive training programmes on perinatal mental health screening and management should be incorporated into both pre-service curricula and continuing professional development for all cadres of healthcare workers. Second, formal referral pathways and collaborative protocols between traditional healers and clinical facilities should be established, building on existing community trust in traditional practitioners. Third, community education campaigns are needed to destigmatise perinatal mental illness and raise awareness of available clinical services, thereby improving care-seeking behaviour among affected women.

### Strengths and limitations

This study’s mixed-methods design represents a key strength, enabling the triangulation of quantitative prevalence data with rich qualitative insights into lived experiences and systemic factors. The use of validated, translated screening instruments (PHQ-9 and GAD-7) and a relatively large community sample enhances the reliability and applicability of the quantitative findings. The purposive inclusion of three stakeholder groups, perinatal mothers, healthcare workers, and traditional healers, provides a multidimensional understanding of the issue.

Several limitations should be acknowledged. First, the study was conducted in a single district in Southern Malawi, which may limit the generalisability of findings to other regions with different cultural or socioeconomic profiles. Second, the cross-sectional design precludes causal inference. Third, PHQ-9 and GAD-7 are validated self-report screening instruments, not diagnostic interviews; they identify probable symptom severity but cannot confirm a clinical psychiatric diagnosis, which would require a structured clinical interview by a trained mental health professional. Throughout this study, ‘moderate-to-severe depressive/anxiety symptoms’ refers to screening-positive status on these instruments, and findings should not be over-interpreted as confirmed disorder prevalence. Responses are also subject to social desirability bias, which may result in under-reporting of symptoms in a stigmatised domain. Furthermore, questionnaires were administered by trained research assistants rather than self-completed, which may have further amplified social desirability bias given the sensitive nature of perinatal mental health. Fourth, the target sample size was slightly reduced from the calculated 384 to 350 for practical reasons, and the final sample of 330 represents a modest shortfall. Fifth, the inclusion criterion requiring participants to have accessed both traditional and clinical care may have introduced selection bias: women with greater mental health needs may be more likely to seek multiple care pathways, potentially inflating the reported prevalence estimates relative to the general perinatal population. Future studies should employ longitudinal designs, larger samples, clinician-administered diagnostic interviews, and broader inclusion criteria to address these limitations.

## Conclusion

This study demonstrates that perinatal depressive and anxiety symptoms represent a significant public health challenge in Southern Malawi, affecting more than one-third of the perinatal women surveyed. Traditional healing practices play a central, culturally validated role in perinatal mental healthcare, yet are not matched by accessible, empathetically delivered, or adequately resourced clinical services. The barriers identified, including stigma, limited provider training, and inadequate infrastructure, are systemic in nature and require coordinated responses at the policy, facility, and community levels.

Integration of traditional and clinical care, underpinned by mutual respect and structured collaboration, offers a viable and culturally grounded pathway to more comprehensive and effective perinatal mental health support. Such integration must be accompanied by investment in healthcare worker capacity building across both assessment and intervention competencies, as well as sustained community engagement. Both traditional healers and clinical providers have distinct and complementary roles to play; neither system alone is sufficient to meet the complex perinatal mental health needs of women in low-resource settings.

The findings of this study are applicable beyond Malawi to other low-resource settings across sub-Saharan Africa where traditional healing is deeply embedded in culture and where clinical mental health infrastructure remains underdeveloped. Empowering perinatal mothers through a genuinely integrated care system, one that honours cultural practice while delivering evidence-based clinical support, is both a moral imperative and a public health priority. Future research should employ longitudinal and comparative designs to build on these findings and to evaluate the effectiveness of integrated care models in improving perinatal mental health outcomes.

## Data Availability

The data that support the findings of this study are available from the corresponding author upon reasonable request.
